# Chemical Composition and Antifungal Activity of *Artemisia sieversiana* Essential Oil Growing in Jilin Against Black Spot on Yanbian Pingguoli Pear in China

**DOI:** 10.3390/plants15020207

**Published:** 2026-01-09

**Authors:** Rong Zhang, Ti-Yan Zheng, Yu Fu

**Affiliations:** Department of Chemistry, Yanbian University, Yanji 133002, China; 15034734254@163.com (R.Z.); 19315219926@163.com (T.-Y.Z.)

**Keywords:** essential oil, antifungal activity, black spot, Pingguoli

## Abstract

Black spot disease substantially impairs both the aesthetic quality and commercial viability of affected Pingguoli pears. Previous studies have shown that *Alternaria alternata* and *A. tenuissima* are the pathogens that cause black spot disease. Essential oils represent novel alternatives to synthetic fungicides to control these pathogens. This study extracted *Artemisia sieversiana* essential oil (AsEO) by hydro-distillation using a crystal tower pure dew essential oil machine. The chemical compositions of AsEO were analyzed via gas chromatography–mass spectrometry (GC–MS). A total of 42 compounds were detected. 1,8-cineole, trans-caryophyllene, (1R,4S)-1,7,7-trimethylbicyclo [2.2.1] heptan-2-yl acetate, (±)-camphor, and β-myrcene were identified as the five main constituents. Moreover, the antifungal activity of AsEO was assessed against black spot on Yanbian Pingguoli pear in China. The minimum inhibitory concentration (MIC) and minimum fungicidal concentration (MFC) values were determined as 0.10% (*v*/*v*) and 0.12% (*v*/*v*), respectively. Scanning electron microscopy (SEM) analysis revealed that treatment with AsEO induced significant morphological aberrations in *A. alternata* and *A. tenuissima* mycelia, including surface roughening, hyphal collapse, and loss of structural integrity. Concurrently, a marked increase in alkaline phosphatase (AKP) enzyme activity and electrical conductivity was observed, a key indicator of cell wall and plasma membrane permeabilization and damage. When the concentration of AsEO was less than 120 µg/mL, there was no toxicity to keratinocytes (HaCaTs) and skin fibroblasts (NHSFs). In summary, this study provides a theoretical basis for the development of AsEO as a fungicide against black spot disease on Pingguoli pear in China.

## 1. Introduction

Members of the *Alternaria* genus, classified within the order Pleosporales (Ascomycota), exhibit a cosmopolitan distribution and function ecologically as both phytopathogens and saprophytes. *Alternaria* comprises over 300 described species that have caused serious economic losses in global horticulture through diseases like black spot and black rot [1]. Normally, *Alternaria* species are categorized into two obviously distinct groups: large-spored and small-spored *Alternaria* species [2]. The conidial bodies of large-spored species typically measure 60–100 μm in length, while the small-spored species are less than 60 μm. Several small-spored species can infect humans and animals, causing diseases such as tinea, onychomycosis, and osteomyelitis of the jawbone [3]. Phylogenetically diverse *Alternaria* species demonstrate remarkable biosynthetic versatility, producing over 150 secondary metabolites with taxonomic significance [4]. This chemical repertoire encompasses: alternariol (AOH), alternariol monomethyl ether (AME), altenuene (ALT), altertoxin I, II, and III (ATXI, II, and III), tenuazonic acid (TEA), iso-tenuazonic acid (iso-TEA), and tentoxin (TEN). AOH and AME exhibit dose-dependent cytotoxicity and genotoxicity at micromolar concentrations, primarily through mechanisms such as DNA intercalation or oxidative stress induction [5]. In contrast, ATXs display markedly enhanced acute toxicity (e.g., lower LD_50_ values in murine models) and mutagenic potential relative to AOH/AME [6]. TEA demonstrates mild cytotoxicity and mutagenicity in vitro, but in vivo studies have shown that it exhibits acute toxicity and synergistic effects in the presence of other toxins [7]. TEN, a phytotoxin known to disrupt chlorophyll synthesis and induce chlorosis in plant seedlings via photosystem II inhibition, remains understudied in mammalian systems [8]. These *Alternaria* toxins are primarily produced by various species, such as *A. alternata*, *A. arborescens*, *A. brassicae*, *A. capsici-anui*, *A. citri*, *A. cucumerina*, *A. dauci*, *A. japonica*, *A. longipes*, *A. mali*, *A. solani*, *A. tenuissima*, and *A. tomato* [9]. Moreover, the metabolic products have been detected in different substrates (tomato, wheat, apple, pear, walnuts, etc.) [9]. Therefore, it is crucial to find effective biological disinfectants.

The taxonomically diverse genus *Artemisia* L. (*Asteraceae*), comprising approximately 500 species globally, exhibits remarkable biogeographical patterns, with significant endemism in China, where 190 taxa have been documented across diverse ecosystems [10]. *Artemisia sieversiana* Ehrhart ex Willd., known as “Da-Zi-Hao” in Chinese, is an annual or biennial herbaceous rhizome plant of the *Artemisia* family that grows in sandy clay, moist habitats, and is distributed in Liaoning, Jilin, Hebei, Shanxi, Gansu, Xinjiang, and Xizang province in China [11]. *A. sieversiana* contains sesquiterpenes, dimeric guaianolides, lignans, flavones, and essential oils [10]. *AsEO* is widely known for its various active properties, such as its antitumor, anti-inflammatory, antiparasitic and antifungal effects [12,13,14]. The components of plant essential oils vary significantly depending on factors such as region, temperature, geographic origin, photoperiod, edaphic influences, season, and microbial diversity [15]. Zhang et al. [10] found that the major components of AsEO growing in Hebei province, China, were neryl propanoate, β-nerol and β-cubebene. Suleimenov et al. [13] found that the principal components of AsEO from plants growing in West Kazakhstan Oblast were myrcene, 1,8-cineol, linalool, p-cymene, nerylisovalerate, and β-caryophyllene. Liu et al. [14] reported that the major chemical components of AsEO from Beijing were eucalyptol, geranyl butyrate, borneol, and camphor. Zhigzhitzhapova et al. [16] found that the principal constituents of AsEO from Buryatia were chamazulene, germacrene D, a-bisabolol, neryl-3-methylbutanoate, and neryl-2-methylbutanoate. Liu et al. [17] reported that the major chemical components of AsEO from Liaoning province, China, were eucalyptol, (–)-borneol, (+)-camphor, β-caryophyllene, and (E)-β-farnesene. Jiang et al. [18] found that the principal constituents of AsEO from Xinjiang, China, were a-thujone, eucalyptol, and santolina triene. Li et al. [19] found the major components of AsEO from Tibet were chamazulene, α-bisabolol, and α-phellandrene. Until now, the major components of AsEO from plants grown in Jilin province, China, has not been studied. Although numerous studies have shown that AsEO has an antifungal effect, little research has been conducted on *Alternaria* species.

Previous research confirmed that the pathogens that cause black spot disease in Yanbian Pingguoli pear were *A. tenuissima* and *A. alternate*. In this study, we used GC-MS to analyze the main chemical components of AsEO. The effect of AsEO on *Alternaria* pathogens was also assessed. These findings are anticipated to generate further scientific interest in the sustainable utilization of *A. sieversiana*.

## 2. Results

### 2.1. Chemical Composition of the AsEO

The blue essential oil was obtained from *Artemisia sieversiana*. Spectral authentication (>99% confidence level) was implemented via multi-dimensional spectral deconvolution of raw GC-MS data with the National Institute of Standards and Technology (NIST) mass spectral library (version 20), complemented by chromatographic co-elution validation using in-house synthesized reference standards. A total of 42 components were found in AsEO, accounting for 98.2% of the total oil (Table 1). The structural formulas of the various components in AsEO are shown in Supplementary Table S1. The main components of the essential oil were 1,8-cineole (34.03%), trans-caryophyllene (22.38%), and (1R,4S)-1,7,7-trimethylbicyclo [2.2.1] heptan-2-yl acetate (8.39%), followed by (±)-camphor (7.89%), and β-myrcene (3.90%). The chemical composition of the essential oil was different from that reported in other studies [17,18,19]. The distinctive chemical profiles of AsEOs extracted from plants growing in different localities could be explained by geographic locality, climate, stress (such as drought), harvest time, etc.

### 2.2. Antifungal Activity of AsEO

**Figure 1:**
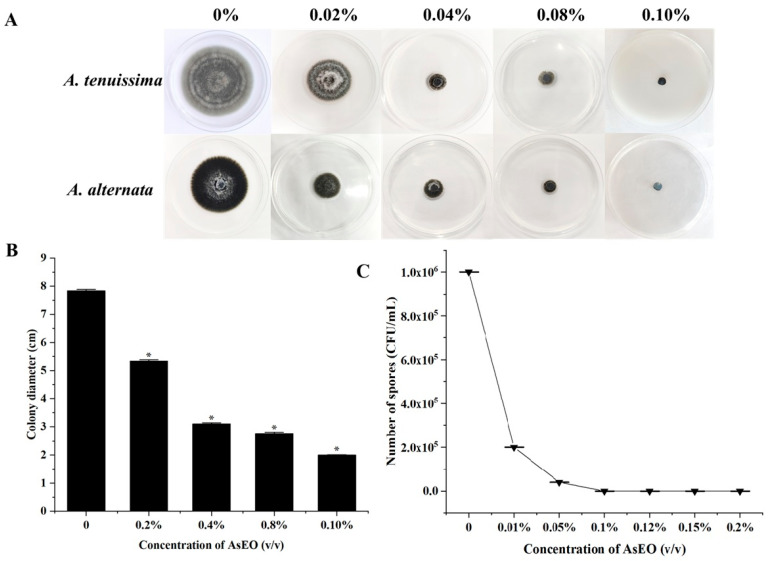

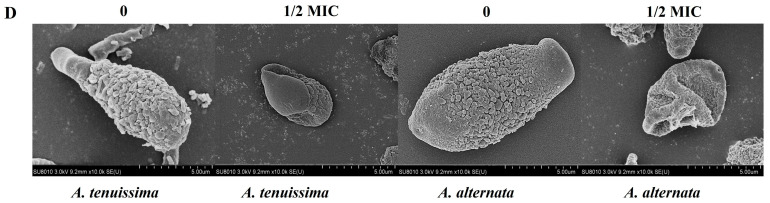
Antifungal activity of AsEO against *A. tenuissima* and *A. alternate*. (**A**) Morphology of *A. tenuissima* and *A. alternate*; (**B**) diameter of *A. tenuissima* colonies after 7 d with different concentrations of AsEO; (**C**) number of A. *tenuissima* spores treated with different concentrations of AsEO in potato dextrose broth (PDB) medium for 3 d; (**D**) SEM of control (0 *v*/*v*) and 1/2 MIC (0.10% *v*/*v*) carvacrol-treated *A. tenuissima* and *A. alternate* spores for 48 h. Values represent the mean ± SD (n = 3; * *p* < 0.05).

As shown in Figure 1A,B, the colony diameter decreased from 7.85 ± 0.04 cm to 2.0 cm as the concentration of AsEO increased. No growth inhibition was observed in control Petri plates. Different concentrations (0.02–0.10%, *v*/*v*) of AsEO had significantly inhibitory effects on lesion diameter. There was no statistically significant difference mycelium growth diameter between *A*. *tenuissima* and *A. alternate* when treated with AsEO. Mycelial growth was completely inhibited when exposed to an AsEO concentration of 0.10% (*v*/*v*), which was defined as the MIC (Figure 1A). The mycelial growth in transferred potato dextrose agar (PDA) plates was completely inhibited when exposed to a concentration of 0.12% (*v*/*v*), which was defined as the MFC (Figure 1C). SEM analysis revealed striking ultrastructural differences between control and AsEO-treated *A. tenuissima* and *A. alternate* (Figure 1D). The control group exhibited intact, smooth hyphal surfaces with uniform cellular outlines, whereas AsEO exposure (1/2 MIC, 48 h) induced severe morphological aberrations: hyphae appeared shrunk, wrinkled, and rough. These alterations are indicative of AsEO’s disruptive effects on fungal cell wall integrity and cytoskeletal organization in *A*. *tenuissima* and *A. alternate*. These results confirm that AsEO has the potential to inhibit black spot disease in Yanbian Pingguoli pear.

### 2.3. AsEO Disrupts Cell Wall Integrity

**Figure 2:**
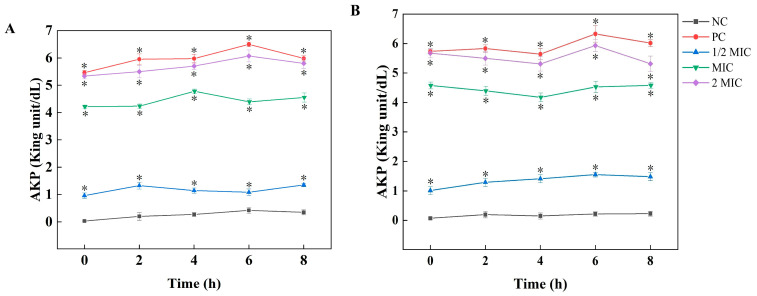
Effects of AsEO on AKP enzyme activity of the pathogenic fungi causing Yanbian Pingguoli pear black spot at 28 °C. (**A**) *A. tenuissima*; (**B**) *A. alternata*. Values represent the mean ± SD (n = 3; * *p* < 0.05).

The structural integrity of microbial cell walls, which serve as protective barriers against environmental stressors, was systematically evaluated through AKP activity profiling. Positioned at the interface between the peptidoglycan layer and cytoplasmic membrane, AKP serves as a sensitive biomarker for cell wall permeabilization. As illustrated in Figure 2, exposure to AsEO induced a time-dependent increase in extracellular AKP activity (R^2^ = 0.96), with a 2 MIC treatment demonstrating a 6.4 ± 0.5-fold elevation compared to controls after 6 h (*p* < 0.001).

### 2.4. AsEO Damages Cell Membrane Integrity

**Figure 3:**
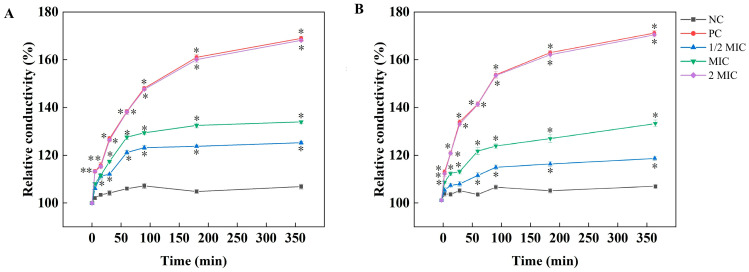
Impact of various concentrations of AsEO (0.5 MIC, MIC, and 2 MIC) on cell membrane integrity in *Alternaria* species. (**A**) *A. tenuissima*; (**B**) *A. alternata*. Values represent the mean ± SD (n = 3; * *p* < 0.05).

The cell membrane is a protective layer, and plays an extremely important role in organism metabolism. Cell membrane damaged, causes imbalances in osmotic pressure and pH, increases the permeability of the cell membrane, leads to electrolyte leakage, and can even cause cell death. Therefore, cell membrane permeability can be evaluated by measuring the conductivity of the cell culture medium. As shown in Figure 3, there was a significant increase in nucleic acid leakage (1.8-fold extracellular conductivity for 360 min at 2 MIC). These results indicate that AsEO is capable of disrupting cell membrane integrity in *A*. *tenuissima* and *A. alternata*.

### 2.5. Safe Concentration of AsEO on Human Keratinocytes Cells (HaCaTs) and Skin Fibroblasts Cells (HSFs)

As shown in Table 2, cell viability under different concentrations of AsEO was determined using the CCK-8 assay. The analysis indicated that AsEO concentrations of less than 120 µg/mL had no effect on the activity of HaCaTs and NHSFs. As the concentration of the essential oil decreased, the safety to cells increased accordingly. At a concentration of 50 µg/mL, the essential oil promoted HaCaT proliferation; within the range of 10 to 120 µg/mL, it significantly promoted NHSF proliferation.

## 3. Discussion

The Yanbian Pingguoli pear (*Pyrus pyrifolia* var. Yanbian Pingguoli pear), a cultivar with distinct apple-like morphology, was first introduced to Yanbian Korean Autonomous Prefecture (Jilin Province, China) through grafting experiments conducted in 1921 by Cui Changhao using scions imported from North Hamgyong Province, DPRK [20]. This historical introduction marked the origin of Asia’s largest Pingguoli pear cultivation base, currently spanning 12,000 hectares with annual production exceeding 90,000 metric tons and generating nearly CNY ¥1 billion in economic value [20]. *Alternaria*-induced black spot disease represents a critical constraint in Yanbian Pingguoli pear cultivation, severely reducing the quality of the fruit’s appearance and its economic value. This foliar pathogen manifests as initial circular, dark brown lesions (1–7 mm diameter) with chlorotic halos, progressing into concentric rings of necrotic tissue (tan to brown-black pigmentation) and culminating in complete leaf abscission. When there are injuries on the trunk and roots of the tree, the pathogen will directly invade the tree tissues. The lesion will spread throughout the tree body, resulting in rotting and causing the tree to become weak or even die. When immature fruit is infected, there are occasionally several gray-brown spots on the surface of the fruit. Gradually, larger turbinate-shaped spots appear on the surface of the fruit, the fruit body begins to sink, and eventually, the fruit rots. Meanwhile, when immature fruits are infected, there are occasionally several gray-brown spots on the surface of the fruits. Subsequently, there is a significant increase in the number and size of black spots on the fruit surface. Large spots appear on the surface of the fruits and the fruit body begins to sink, eventually leading to fruit rotting. In the late stages of storage, the incidence of black spot disease is quite high, reaching 37% [21]. Chemical fungicides exhibit superior efficacy in limiting the dissemination of fungal phytopathogens and soil-borne infections due to their rapid biocidal action and environmental persistence. However, the excessive use of chemical disinfectants not only leads to the development of drug resistance in pathogenic fungi, but also causes environmental pollution [22]. Consequently, the development of novel, eco-friendly fungicides has become a critical priority in combating *Alternaria* species and their associated mycotoxin contamination.

Plant essential oils are aromatic secondary metabolites derived from plant materials. They exhibit a broad range of sources and chemical diversity, and demonstrate various biological activities, including antibacterial [23], anti-inflammatory [24], and antioxidant properties [25]. In recent years, extensive domestic and international research has demonstrated that plant essential oils possess significant antifungal efficacy against a wide spectrum of pathogenic fungi, positioning them as promising alternatives to conventional fungicides [26,27,28]. However, due to their high variability in composition and structural complexity, the underlying antibacterial mechanisms of these essential oils can differ substantially. Therefore, identifying the bioactive constituents responsible for antimicrobial activity and elucidating the modes of action of plant essential oils are crucial steps toward the development of effective natural antimicrobial agents. The extraction methods for plant essential oils include distillation, pressing, ultrasonication, microwave treatments, and ohmic heating [29]. Previous studies have all extracted AsEO via the steam distillation method [10,13,16,17,18,19]. *A. sieversiana* was subjected to hydro-distillation using a modified Clevenger-type apparatus for 2–6 h, with the extract quantity at 17–43. In this study, we first used a crystal tower pure dew essential oil machine (25L-DW, Hangzhou Hooloo Industrial Co., Hangzhou, China) for 4 h, and 42 compounds were obtained. Taking into account yield and distillation time, using a crystal tower pure dew essential oil machine could be an excellent approach for extracting essential oils.

Phytochemical analysis revealed that plant essential oils (EOs) exert antimicrobial effects through multiple mechanisms, including the cell walls and membrane integrity disruption, subcellular morphological alterations, nucleic acid impairment, enzymatic inhibition, and metabolic pathway perturbation [30]. Phylogenetic analyses reveal *A. tenuissima* and *A. alternata* as the primary etiological agents, implicated in black spot disease in Yanbian Pingguoli pear [20]. Many components of AsEOs play significant roles in the antifungal and antioxidant properties of the oils. 1,8-Cineole is a potential substitute for synthetic fungicides against *Alternaia tenuissima* [31]. Camphor can significantly inhibit fungal growth [32]. trans-Caryophyllene and β-myrcene (identified as predominant compounds in AsEO) are good indicators of antioxidant efficiency [33]. Terpenoids (such as limonene, linalool, etc.) and aromatic compounds (such as eugenol, cinnamaldehyde, etc.), identified as predominant compounds in AsEO, can disrupt membranes structure through hydrophobic interactions [34]. The antifungal properties of the main components of the AsEO were verified in this study. Meanwhile, the integrity of fungal membrane systems could be quantitatively assessed through dual-parameter analysis involving extracellular AKP activity and ionic flux dynamics. In the study, exposure to AsEO increased AKP activity and electrical conductivity. The enhanced antifungal activity of AsEO was attributed to its dual-target mechanism, which simultaneously induces biochemical destabilization of fungal cell walls and structural disintegration of membranes.

## 4. Materials and Methods

### 4.1. Collection of Plant Materials

*Artemisia sieversiana* was grown in Yanji (42°54′32″ N, 129°28′47″ E), Jilin Province, China. The fresh aerial biomass (terminal branches with inflorescences) was harvested during the late vegetative phase (15 August 2023), ensuring optimal phenological representation. Specimen authentication was conducted by Professor Dong Weiwei (College of Agriculture, Yanbian University, Yanji, China) utilizing morphological characteristics and molecular barcoding.

### 4.2. Extraction and Isolation of AsEO

The plant materials (2.0 kg) were cut into 3–5 cm pieces, and transferred into crystal tower pure dew essential oil machine (25L-DW, Hangzhou Hooloo Industrial Co., Hangzhou, China). An amount of 4.0 L distilled water and defoaming agent (50 ppm of CaCO_3_) was added, and the distillation rate was set at 25 mL/min. Then, the essential oil was obtained by hydrodistillation for 3 h. Moisture was removed from the final essential oil samples (anh. MgSO_4_), which were stored in airtight containers at 4 °C.

### 4.3. GC-MS Analysis

The chemical compositions of the AsEO were evaluated via GC-MS on a Shimadzu QP 2010 Ultra system (Shimadzu, Tokyo, Japan) equipped with a DB-5MS column (30 m × 0.25 mm ID, 0.25 µm film thickness). The operating conditions were as follows [20]: the initial temperature was 45 °C for 4 min, and then the temperature increased 280 °C at a rate of 6 °C/min, and finalized with a 15 min isothermal hold. The GC-MS system was configured with injector/detector temperatures maintained at 280 °C. Ultra-high-purity helium (≥99%, 0.98 mL/min) served as the carrier gas, with splitless injection (2.0 μL) optimized for volatile compound analysis. Mass spectral acquisition utilized EI ionization (70 eV) with a scan range of 35–500 *m*/*z*. Compound identification integrated NIST 20 spectral library matching (similarity > 85%) against reference standards.

### 4.4. Fungal Strain and Culture Condition

The fungal pathogen species used in this study were *A. tenuissima* (NCBI GenBank Accession No: OQ001067) and *A*. *alternata* (NCBI GenBank Accession No: OQ001055), previously isolated from black spot disease on Yanbian Pingguoli pear [21]. The fungal species were subcultured on PDA medium, which was supplemented with 100 μg/L of streptomycin sulfate and incubated for 7 days at 28 °C. PDA medium: 200 g of potato, 15 g of agar powder, mixed with 1000 mL of deionized water, sterilized under high pressure at 121 °C for 25 min, placed upright, and cooled to 40 °C before pouring into disposable Petri dishes and left to stand for later use. The fungal pathogen species were cultured to logarithmic growth stage on potato dextrose Broth (PDB) medium. PDB medium: 200 g of potato, mixed with 1000 mL of deionized water, sterilized under high pressure at 121 °C for 25 min, cooled to room temperature and stored.

### 4.5. Antifungal Effect of the AsEO on Alternaria Species

The antifungal effect of AsEO was performed following the method described by Kou et al. [20]. The five concentrations of AsEO (*v*/*v*) used were 0%, 0.02%, 0.04%, 0.08%, and 0.10%. After incubation at 28 °C for 7 days, colony diameters were measured using the crossover approach to determine the percentage of inhibition for each concentration of AsEO treatment. The MIC was determined as the lowest concentration of the AsEO at which no visual fungal growth was observed. To determine the MFC of the AsEO, 10 μL of the AsEO (at the MIC and higher concentrations) was transferred to PDA plates. The plates were incubated for 48–72 h at 28 °C. The lowest concentration at which the fungus had no growth was considered as the MFC.

Spore samples were cultured on PDB medium supplemented with 0 and 1/2 MIC of AsEO, followed by sample collection for SEM analysis. The samples were fixed in 2.5% glutaraldehyde at 4 °C for 48 h and dehydrated using the method described by Kou et al. [20]. After dehydration, samples were mounted on the SEM stage, coated with gold for 60 s, and examined using SEM (SU-8010; Hitachi, Tokyo, Japan).

### 4.6. Effect of the AsEO on Cell Wall

The fungal pathogen species were cultured to the logarithmic growth stage on PDB medium, and different volume fractions of the AsEO were added to the spore suspension to achieve final AsEO volume fractions of 0.5 MIC, MIC, and 2 MIC. The fungal suspensions of *A. tenuissima* and *A. alternata* were 1 × 10^6^ CFU/mL. The negative control (NC) group received an equivalent volume of ddH_2_O, and was set at 100%, while the positive control (PC) group was treated with a lethal concentration of carbendazim solution. All experimental groups were incubated in a shaker at 37 °C and 120 rpm for 8 h. Samples were taken at 0 h, 2 h, 4 h, 6 h, and 8 h. After low-temperature centrifugation at 4 °C and 10,000 rpm for 10 min, the supernatants were collected and processed according to the instructions in the AKP kit (Beijing Bioss Technology Co., Beijing, China). Absorbance at 510 nm was measured using a UV-2550 ultraviolet–visible spectrophotometer (Shimadzu, Tokyo, Japan). The amount of phenol produced per gram of tissue per minute at 37 °C was taken as 1 μmol. The experiment was conducted in triplicate. Subsequently, a time-course kinetic profile was construct with reaction time (x-axis, 0–8 h) and AKP activity (y-axis, U/L) to analyze the effect of the AsEO on the cell walls of the two fungal pathogens.

The calculation formula for the AKP content in *Alternaria* species is as follows:

(1) $$AKP=\frac{A1-A0}{A2-A0}\times a\times b$$

where A1 is the absorbance of the sample, A0 is the absorbance of the blank, A2 is the absorbance of the standard sample, a is the concentration of phenol standard sample, and b is the dilution factor.

### 4.7. Effect of the AsEO on Cell Membrane

Electrical conductivity was assessed based on the foundational methodology of Min et al. [35]. A 200 μL aliquot of 1 × 10^6^ CFU/mL spore suspensions of *A. tenuissima* and *A. alternata* were cultured in PDB at 28 °C for 4 days, after which grayish-black hyphae was collected. Subsequently, 0.5 g of hyphae sample was incubated in 5.0 mL distilled water with varying concentrations of AsEO (0.5 MIC, MIC, and 2 MIC) as the experimental groups; The negative and positive control groups were as previously described. Conductivity was measured at 0, 5, 15, 30, 60, 90, 180, and 360 min using a DDSJ-308F conductivity meter (Leizi, Shanghai, China). Next, the solution was boiled in water for the sterilization. After the solution cooled to room temperature, the conductivity was measured again. The experiment was repeated three times. The relative conductivity at each time point was calculated using the conductivity formula. Variance analysis (*p* < 0.05) was completed using Graphpad Prism 7 software. To verify the effect of the AsEO on cell membrane permeability in *Alternaria* species, the trend graphs for each group were plotted. The culture time was on the x-axis and the conductivity was on the y-axis.

The formula for calculating relative conductivity is as follows:

(2) $$C=\frac{C1-C0}{C2}\times 100\%$$

where C is the relative conductivity (%), C1 is the electric conductivity at a certain time, C0 is the initial electric conductivity, and C2 is the final electric conductivity.

### 4.8. Determining the Safe Concentration of the AsEO

The safe concentration of the AsEO was determined using the cell counting kit-8 assay (Merck KGaA, Darmstadt, Germany). Human immortalized keratinocytes HaCaTs (BNCC101683) and skin fibroblasts HSFs (BNCC338008) were purchased from Beijing Beina Biotechnology Co., Ltd., Beijing, China. The HaCaT and HSF concentrations were adjusted to 5 × 10^3^ CFU/mL, before inoculating cells into 96-well culture plates. A 100 µL aliquot of the cell suspension was added to each well. After 24 h of cultivation, a 50 µL aliquot of the culture medium containing different concentrations (0.1, 1, 10, 50, 100, 120, 150, 500, 1000 µg/mL) of the AsEO were added to each well. Control group (without adding AsEO) and blank groups (only culture medium, without cells) were also established. Each group had 4 duplicate wells. The absorbance (A value) at 450 nm was determined using the cell counting kit-8 [36].

The calculation formula for relative cell viability is as follows:

(3) $$A=\frac{As-Ab}{Ac-Ab}\times 100\%$$

where A represents the relative cell viability (%), As represents the absorbance values of the cells containing different concentrations of the AsEO and the experimental wells containing the cell counting kit-8 medium. Ac represents the absorbance values of the control wells containing only cells and the cell counting kit-8 medium, and Ab represents the absorbance values of the blank wells containing only the cell counting kit-8 medium.

### 4.9. Statistical Analysis

All experimental procedures were performed in three independent replicates, with quantitative outcomes presented as mean values ± standard deviation (SD). Comparative analysis between experimental groups was conducted using Student’s *t*-test (α = 0.05), while parametric ANOVA analysis with Bonferroni post hoc correction was applied for multiple group comparisons. Statistical processing was performed using Statistical Package for the Social Sciences (SPSS) version 25.0 (IBM Corporation, Armonk, NY, USA), with significance thresholds set at *p* < 0.05.

## 5. Conclusions

In this study, the essential oil extracted from *Artemisia sieversiana* (Jilin biotype) was subjected to comprehensive GC-MS analysis with electron ionization (EI 70 eV) and a scan rate of 3 scans/s. A total of 42 bioactive volatiles were identified through NIST 20 spectral matching (similarity > 85%), with the key constituents being 1,8-cineole (34.03%), trans-caryophyllene (22.38%), (1R,4S)-1,7,7-trimethylbicyclo [2.2.1] heptan-2-yl acetate (8.39%), (±)-camphor (7.89%), and β-myrcene (3.90%). These bioactive constituents exhibited concentration-dependent antifungal activity against *A*. *tenuissima* and *A. alternate*, the causal agents of black spot disease in Yanbian Pingguoli pear. The MIC and MFC values were determined as 0.10% (*v*/*v*) and 0.12% (*v*/*v*), respectively. AsEO exhibited concentration-dependent cytoplasmic membrane permeabilization and cell wall degradation in *A*. *tenuissima* and *A. alternate*. This dual-target mechanism positions AsEO as a candidate biocontrol agent for managing *Alternaria*-induced black spot disease in Yanbian Pingguoli pear orchards. Further research is warranted to systematically assess the application of AsEO on fruits crops to optimize its biocontrol efficacy during both the growth and storage periods.

## Figures and Tables

**Table 1:** Chemical composition of essential oils extracted from *Artemisia sieversiana*.

No.	Compound	Retention Time (min)	Molecular Formula	CAS	Relative Content (>0.10%)
1	α-thujene	10.00	C_10_H_16_	2867-05-2	0.33
2	α-pinene	10.23	C_10_H_16_	80-56-8	1.98
3	(±)-camphor	10.94	C_10_H_16_O	464-48-2	7.89
4	sabinene	11.90	C_10_H_16_	3387-41-5	1.97
5	β-pinene	12.06	C_10_H_16_	127-91-3	0.52
6	Oct-1-en-3-ol	12.27	C_8_H_16_O	3391-86-4	0.19
7	β-myrcene	12.63	C_10_H_16_	123-35-3	3.90
8	2-carene	13.14	C_10_H_16_	554-61-0	1.67
9	δ-4-carene	13.66	C_10_H_16_	29050-33-7	0.79
10	o-cymene	13.99	C_10_H_14_	527-84-4	0.46
11	(+)-limonene	14.15	C_10_H_16_	5989-27-5	0.22
12	1,8-cineole	14.37	C_10_H_18_O	470-82-6	34.03
13	γ-terpinene	15.32	C_10_H_16_	99-85-4	0.55
14	isoterpinolene	16.38	C_10_H_16_	586-63-0	0.42
15	linalool	17.05	C_10_H_18_O	78-70-6	1.98
16	nonanal	17.21	C_9_H_18_O	124-19-6	0.13
17	neo-alloocimene	18.07	C_10_H_16_	7216-56-0	0.10
18	2,4-hexadienyl isobutyrate	18.56	C_10_H_16_O_2_	16491-24-0	0.12
19	cis-chrysanthenol	19.46	C_10_H_16_O	55722-60-6	0.42
20	terpinine-4-ol	20.12	C_10_H_18_O	562-74-3	0.54
21	α-terpineol	20.66	C_10_H_18_O	10482-56-1	0.82
22	(1R,4S)-1,7,7-trimethylbicyclo [2.2.1]heptan-2-yl acetate	20.90	C_12_H_20_O_2_	92618-89-8	8.39
23	decanal	21.04	C_10_H_20_O	112-31-2	0.11
24	nerol	21.69	C_10_H_18_O	106-25-2	0.10
25	cis-3-hexenyl 2-methylbutanoate	22.09	C_11_H_20_O_2_	53398-85-9	0.11
26	benzyl isobutanoate	24.23	C_11_H_14_O_2_	103-28-6	0.10
27	2,4-decadienal	25.02	C_10_H_16_O	2363-88-4	0.11
28	α-amorphene	26.71	C_15_H_24_	20085-19-2	0.11
29	α-copaene	26.94	C_15_H_24_	3856-25-5	0.14
30	β-bourbonene	27.21	C_15_H_24_	5208-59-3	0.47
31	β-elemene	27.40	C_15_H_24_	515-13-9	0.96
32	cis-jasmone	27.49	C_11_H_16_O	488-10-8	0.11
33	trans-caryophyllene	28.43	C_15_H_24_	87-44-5	22.38
34	(E)-β-famesene	29.42	C_15_H_24_	18794-84-8	0.15
35	α-caryophyllene	29.54	C_15_H_24_	6753-98-6	0.12
36	cedrene	29.69	C_15_H_24_	11028-42-5	0.10
37	β-selinene	30.61	C_15_H_24_	17066-67-0	0.53
38	(+)-7-epi-sesquithujene	30.77	C_15_H_24_	159407-35-9	0.68
39	farnesene	31.07	C_15_H_24_	502-61-4	1.92
40	δ-cadinene	31.48	C_15_H_24_	483-76-1	0.31
41	methyl isocostate	35.12	C_16_H_24_O_2_	132342-55-3	1.97
42	cembrene	39.49	C_20_H_32_	1898-13-1	1.10

**Table 2:** The survival rates of different cells using CCK-8 assay.

Group	HaCaT	NHSF
1000 μg/mL	28.16 ± 2.15	12.82 ± 0.98
500 μg/mL	55.48 ± 1.18	20.44 ± 1.36
150 μg/mL	60.45 ± 3.28	54.16 ± 5.26
120 μg/mL	95.22 ± 4.58	102.24 ± 7.15
100 μg/mL	102.12 ± 2.36	106.18 ± 3.22
50 μg/mL	92.01 ± 1.98	118.36 ± 3.24
10 μg/mL	88.26 ± 1.08	98.04 ± 4.21
1 μg/mL	86.62 ± 1.26	99.87 ± 4.17
0.1 μg/mL	87.34 ± 3.17	109.38 ± 3.43
*p* value	0.03	0.04

## Data Availability

The original contributions presented in this study are included in the article/supplementary material. Further inquiries can be directed to the corresponding author.

## Supplementary Materials

The following supporting information can be downloaded at: https://www.mdpi.com/article/10.3390/plants15020207/s1, Table S1: Structural formulas of various components in the essential oil from *Artemisia sieversiana*.
