# Supplementary material for: Chemical Composition and Antifungal Activity of Artemisia sieversiana Essential Oil Growing in Jilin Against Black Spot on Yanbian Pingguoli Pear in China
Source: Plants (Basel). 2026 Jan 9;15(2):207. doi: 10.3390/plants15020207 (PMC12845028; doi:10.3390/plants15020207)
Supplement: Supplementary file 1 [file plants-15-00207-s001.zip › plants-4005012-supplementary.pdf]

Supplementary Table S1 Structural formulas of various components in the essential oil from *Artemisia sieversiana.*

No.	Compound	Retention time (min)	Molecular formula	CAS	Structural formulas
1	α-thujene	10.00	$C_{10}H_{16}$	2867-05-2	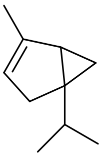
2	α-pinene	10.23	$C_{10}H_{16}$	80-56-8	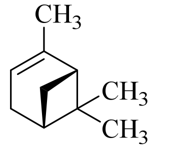
3	(±)-camphor	10.94	$C_{10}H_{16}O$	464-48-2	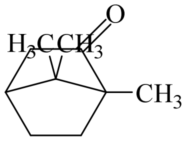
4	sabinene	11.90	$C_{10}H_{16}$	3387-41-5	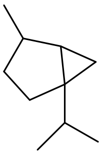
5	β-pinene	12.06	$C_{10}H_{16}$	127-91-3	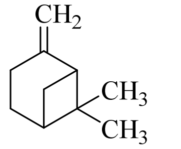
6	Oct-1-en-3-ol	12.27	$C_8H_{16}O$	3391-86-4	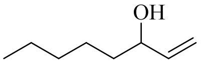
7	β-myrcene	12.63	$C_{10}H_{16}$	123-35-3	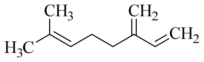
8	2-carene	13.14	$C_{10}H_{16}$	554-61-0	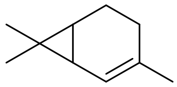
9	δ-4-carene	13.66	$C_{10}H_{16}$	29050-33-7	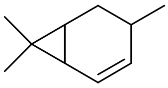
10	o-cymene	13.99	$C_{10}H_{14}$	527-84-4	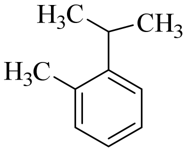
11	(+)-limonene	14.15	$C_{10}H_{16}$	5989-27-5	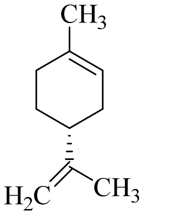
12	1,8-cineole	14.37	$C_{10}H_{18}O$	470-82-6	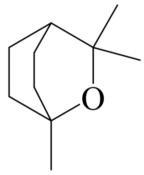

13	γ-terpinene	15.32	$C_{10}H_{16}$	99-85-4	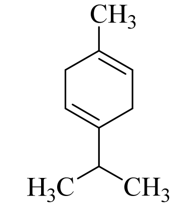
14	isoterpinolene	16.38	$C_{10}H_{16}$	586-63-0	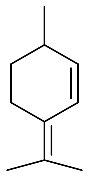
15	linalool	17.05	$C_{10}H_{18}O$	78-70-6	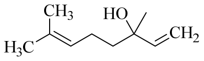
16	nonanal	17.21	$C_9H_{18}O$	124-19-6	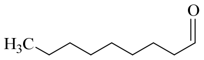
17	neo-alloocimene	18.07	$C_{10}H_{16}$	7216-56-0	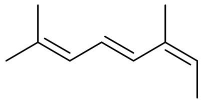
18	2,4-hexadienyl isobutyrate	18.56	$C_{10}H_{16}O_2$	16491-24-0	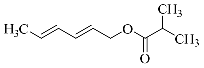
19	cis-chrysanthenol	19.46	$C_{10}H_{16}O$	55722-60-6	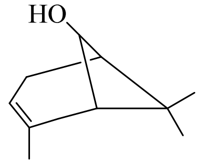
20	terpinine-4-ol	20.12	$C_{10}H_{18}O$	562-74-3	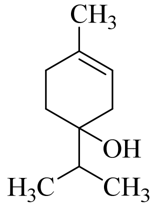
21	α-terpineol	20.66	$C_{10}H_{18}O$	10482-56-1	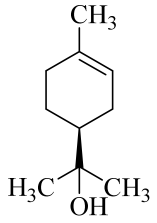
22	(1R,4S)-1,7,7-trimethylbicyclo[2.2.1]heptan-2-yl acetate	20.90	$C_{12}H_{20}O_2$	92618-89-8	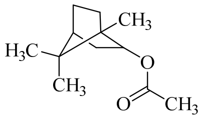
23	decanal	21.04	$C_{10}H_{20}O$	112-31-2	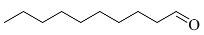
24	nerol	21.69	$C_{10}H_{18}O$	106-25-2	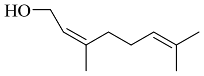
25	cis-3-hexenyl 2-methylbutanoate	22.09	$C_{11}H_{20}O_2$	53398-85-9	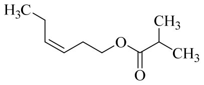
26	benzyl isobutanoate	24.23	$C_{11}H_{14}O_2$	103-28-6	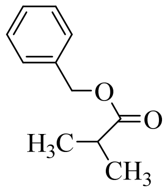
27	2,4-decadienal	25.02	$C_{10}H_{16}O$	2363-88-4	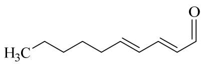

28	α-amorphene	26.71	$C_{15}H_{24}$	20085-19-2	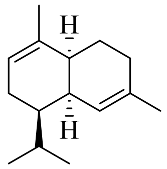
29	α-copaene	26.94	$C_{15}H_{24}$	3856-25-5	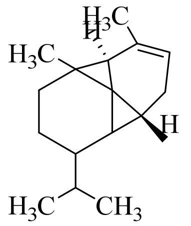
30	β-bourbonene	27.21	$C_{15}H_{24}$	5208-59-3	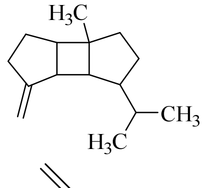
31	β-elemene	27.40	$C_{15}H_{24}$	515-13-9	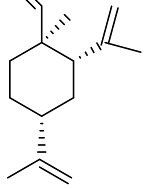
32	cis-jasmone	27.49	$C_{11}H_{16}O$	488-10-8	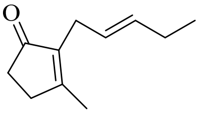
33	trans-caryophyllene	28.43	$C_{15}H_{24}$	87-44-5	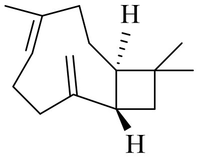
34	(E)-β-famesene	29.42	$C_{15}H_{24}$	18794-84-8	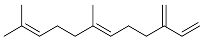
35	α-caryophyllene	29.54	$C_{15}H_{24}$	6753-98-6	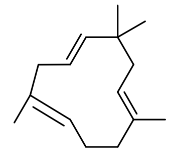
36	cedrene	29.69	$C_{15}H_{24}$	11028-42-5	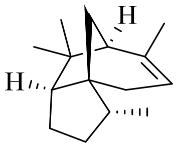
37	β-selinene	30.61	$C_{15}H_{24}$	17066-67-0	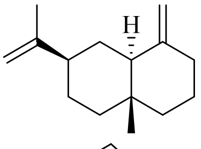
38	(+)-7-epi-sesquithujene	30.77	$C_{15}H_{24}$	159407-35-9	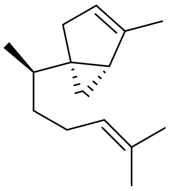
39	farnesene	31.07	$C_{15}H_{24}$	502-61-4	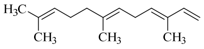

40	δ-cadinene	31.48	$C_{15}H_{24}$	483-76-1	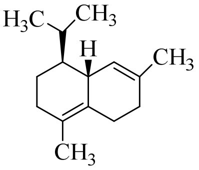
41	methyl isocostate	35.12	$C_{16}H_{24}O_2$	132342-55-3	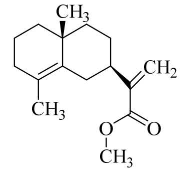
42	cembrene	39.49	$C_{20}H_{32}$	1898-13-1	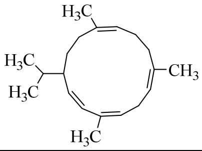
